# Long-Lasting Palliation of Bone Oligometastatic Prostate Cancer After Repeated Stereotactic Body Radiotherapy

**DOI:** 10.31486/toj.20.0132

**Published:** 2021

**Authors:** Ernesto Maranzano, Fabio Arcidiacono, Michelina Casale, Antonella Giannantoni, Nicodemo Baffa, Paola Anselmo, Alessandro Di Marzo, Fabio Trippa

**Affiliations:** ^1^ Radiotherapy Oncology Centre, Hospital Santa Maria, Terni, Italy; ^2^ Department of Medical and Surgical Science and Neuroscience, Functional and Surgical Urology Unit, University of Siena, Siena, Italy; ^3^ Department of PET-CT and Radiological and Laboratory Imaging, Hospital Santa Maria della Misericordia, Perugia, Italy

**Keywords:** *Bone metastases*, *oligometastatic disease*, *prostate cancer*, *stereotactic body radiotherapy*

## Abstract

**Background:** Oligometastatic disease has emerged as a distinct clinical state, with a tumor burden intermediate between localized and extensive systemic disease. Oligometastatic prostate cancer has generally been classified as ≤3 metastases in bone or lymph nodes only. Improvements in diagnostic modalities such as functional imaging allow a greater frequency of oligometastases diagnosis. Selected bone oligometastatic prostate cancer patients can be treated with metastasis-directed stereotactic body radiotherapy (SBRT) rather than androgen deprivation therapy (ADT). We describe a case representative of this scenario.

**Case Report:** A 72-year-old male underwent surgery and salvage radiotherapy for a Gleason score 7 (3+4) adenocarcinoma confined in the prostate but with microscopic-positive surgical margins. Eight months after the end of radiotherapy, bone metastasis was diagnosed and treated with SBRT only because the patient refused ADT. In the subsequent 10 years, 6 more courses of SBRT were administered for new bone oligometastases encountered during follow-up. Neither local recurrence nor toxicity was observed after SBRT treatments. The patient, who is now 83 years old, has a Karnofsky Performance Status score of 90% and has preserved a satisfactory potentia coeundi.

**Conclusion:** SBRT is a promising treatment for patients with bone oligometastatic prostate cancer, providing a high control rate within the irradiated volume and low toxicity. The ability to administer consecutive SBRT courses when new bone oligometastases are encountered in other sites can delay initiation of ADT. This case report reflects emerging trends for bone oligometastases treatment with metastasis-directed radiotherapy.

## INTRODUCTION

Androgen deprivation therapy (ADT) is the basis of treatment for patients with metastatic prostate cancer.^1^ Chemotherapy and hormone therapy with abiraterone or enzalutamide are the second-line pharmacologic approaches for castration-resistant patients.^2^ For castration-naïve metastatic patients with a high volume of metastases (ie, visceral metastases and/or ≥4 bone lesions, with ≥1 bone lesion beyond vertebral bodies and the pelvis) and presentation with de novo metastatic disease, docetaxel can be considered a first-line approach.^3^ In 2017, 2 trials investigated the addition of abiraterone to ADT in high-risk patients who had at least 2 of the following 3 variables: visceral metastasis, ≥3 bone metastases, or a Gleason score ≥8. Both trials showed a decrease of the relative risk of death.^4,5^

Oligometastatic disease has emerged as a distinct clinical state, with a tumor burden intermediate between localized and extensive systemic disease. Although no consensus has been reached regarding the maximum number of metastases that defines oligometastatic disease, oligometastatic prostate cancer has generally been classified as ≤3 metastases in bone or lymph nodes only.^6^ Patients with oligometastatic disease and a controlled primary tumor can be effectively treated with local therapies such as surgery or metastasis-directed radiotherapy. Stereotactic body radiotherapy (SBRT) is the best radiation technique for oligometastatic disease because external beam–accelerated, hypofractionated doses can be administered to the tumor, sparing surrounding healthy tissues by a rapid fall of dose outside the target.^6,7^

Studies published between 2013 and 2019 reported that selected patients with bone metastases from prostate cancer had high local control rates after SBRT and good clinical outcomes without relevant side effects.^8-11^ Moreover, in castration-sensitive patients with prostate cancer bone oligometastases, no sure improvement in outcomes was registered with the addition of ADT to SBRT.^11^ Subsequent courses of SBRT treatment provided to patients with ≤3 new oligometastases encountered during follow-up can be associated with prolonged ADT-free survival (ie, the time interval between the first day of SBRT and the initiation of ADT).^7^ We present a case of long-lasting control of prostate cancer bone oligometastases treated with repeated SBRT without ADT.

## CASE REPORT

In November 2009, a 72-year-old male with a history of arterial hypertension was found to have an elevated prostate-specific antigen (PSA) level of 4.7 ng/mL. He had a localized Gleason score 7 (3+4) prostate adenocarcinoma and chose to undergo radical prostatectomy that confirmed histology: confinement within the prostate, absence of metastases in resected lymph nodes, and microscopic-positive surgical margins. Three months after surgery, the patient's postoperative PSA level was 1.02 ng/mL; computed tomography (CT) and bone scintigraphy were negative for metastases. One month later, salvage intensity-modulated radiotherapy to the prostate bed was administered at a dose of 79.2 Gy in 44 fractions.

On follow-up, the patient did not report any significant bowel or urinary adverse events. His PSA level decreased to 0.7 ng/mL but then increased to 2.34 ng/mL 6 months later. Choline positron emission tomography/CT (F-18 choline PET/CT) demonstrated 1 small focus of increased radiotracer uptake in the left sacroiliac bone consistent with oligometastatic prostate cancer.

ADT, as the standard-of-care treatment option for metastatic prostate cancer, was proposed. The patient refused ADT because of possible iatrogenic-associated sexual impotence. Despite surgery and prostate bed radiotherapy, the patient had preserved urinary continence and satisfactory potentia coeundi. Although few studies had been published about SBRT for prostate cancer bone metastases when treatment options were discussed, SBRT was suggested in an attempt to delay ADT. The patient, a retired medical doctor, was informed about the paucity of scientific evidence supporting this approach. In October 2010, the patient's left sacroiliac bone metastasis was treated with 40 Gy in 5 fractions. Radiotherapy was completed without complication or toxicity. During the next several months, his PSA levels continued to decrease to a nadir of 0.10 ng/mL.

**Figure 1.**
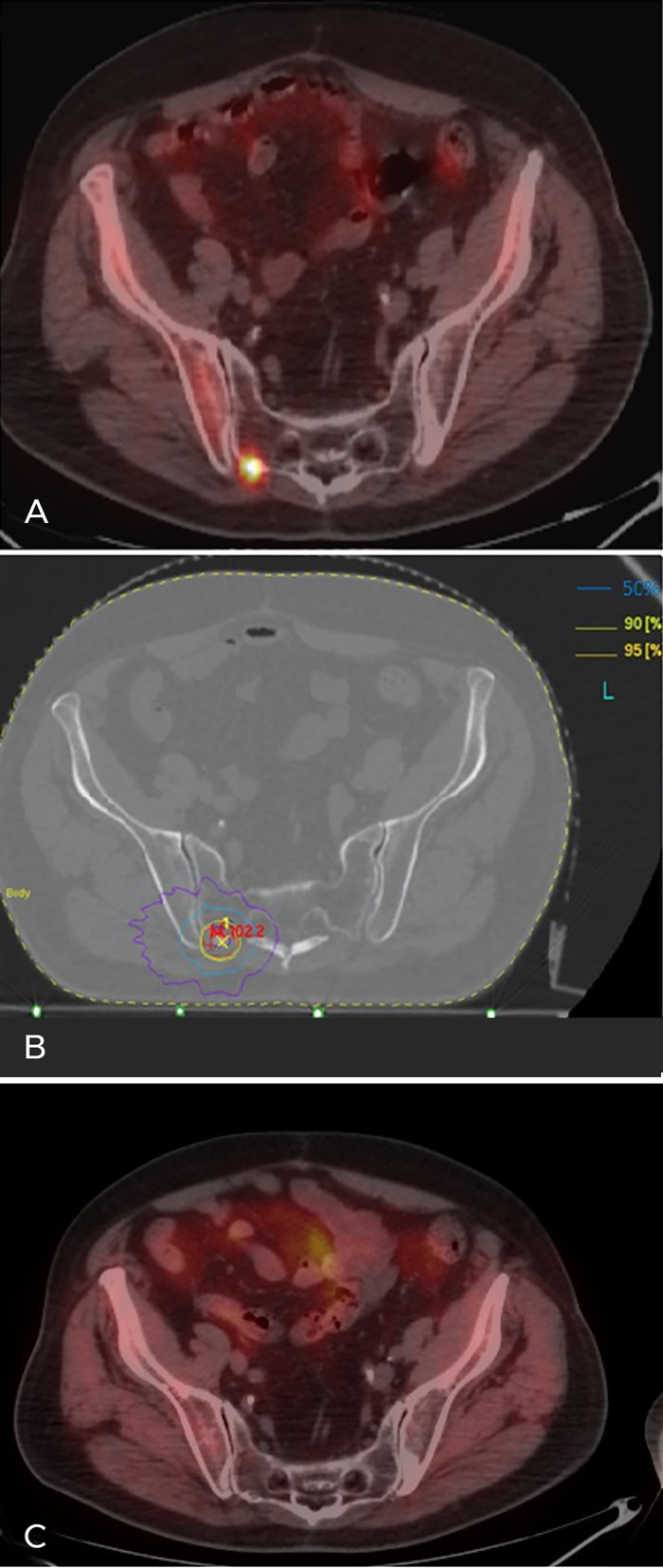
**(A) F-18 choline positron emission tomography/computed tomography (PET/CT) imaging shows right sacroiliac bone metastasis corresponding to the small focus of increased radiotracer uptake. (B) Metastasis-directed stereotactic body radiotherapy was administered at 40 Gy in 5 fractions; isodoses of 95%, 90%, 50%, and 30% (yellow, green, light blue, and violet lines, respectively) indicate the small irradiated volume (isodose of 95%) and the rapid fall of dose outside the target (isodose of 30%). (C) F-18 choline PET/CT imaging showing no radiotracer uptake to the right sacroiliac bone metastasis 10 months after irradiation.** (A color version of this figure is available at https://doi.org/10.31486/toj.20.0132.)

In December 2014, 50 months after the patient's first SBRT, his PSA level began to rise, and new F-18 choline PET/CT imaging demonstrated increased radiotracer uptake in the right sacroiliac joint with an absence of pain (Figure 1). The new metastatic site was treated with SBRT at a dose of 40 Gy in 5 fractions. The patient's PSA level progressively diminished to 0.15 ng/mL.

In July 2016, 19 months after the second course of SBRT, the patient's PSA level increased slightly from 0.15 to 0.35 ng/mL. F-18 choline PET/CT showed positivity in the right iliac wing that was treated with SBRT at a dose of 30 Gy in 3 fractions.

Follow-up continued with 3-month PSA evaluations. Twelve and 22 months later, F-18 choline PET/CT imaging identified 2 new metachronous pelvic bone oligometastatic lesions. Each lesion was treated with SBRT at a dose of 30 Gy in 3 fractions. Neither pain nor toxicity was reported.

Approximately 10 years after prostate surgery and 9 years after his first SBRT treatment for bone metastasis, the patient repeated F-18 choline PET/CT imaging because his PSA levels increased from 0.10 to 0.30 ng/mL. Restaging confirmed the complete radiologic and metabolic response of all previously treated bone sites, but a new oligometastatic location at the anterior region of the fourth thoracic vertebra was identified. SBRT was administered at a dose of 25 Gy in 5 fractions to the entire vertebral body and 35 Gy in 5 fractions to the small focus of increased uptake using the simultaneous integrated boost technique.

**Figure 2.**
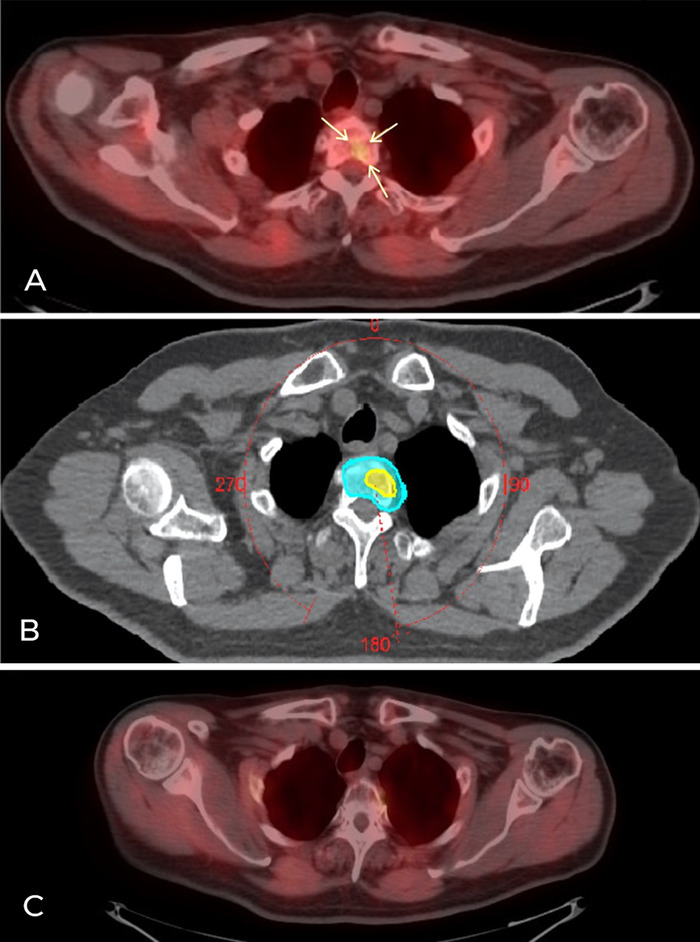
**(A) F-18 choline positron emission tomography/computed tomography (PET/CT) imaging shows bone metastasis in the second thoracic vertebra with a small focus of increased radiotracer uptake (arrows). (B) Metastasis-directed stereotactic body radiotherapy volumes: the light blue shadow is the volume irradiated at 25 Gy in 5 fractions; the yellow shadow denotes the volume corresponding to radiotracer uptake that received 35 Gy in 5 fractions with the simultaneous integrated boost technique. (C) F-18 choline PET/CT imaging shows no radiotracer uptake to the second thoracic vertebra metastasis 8 months after irradiation.** (A color version of this figure is available at https://doi.org/10.31486/toj.20.0132.)

In February 2020, 11 months after his last SBRT, the patient's PSA level increased from 0.13 to 0.57 ng/mL. SBRT was administered where F-18 choline PET/CT identified a small metastasis: to the second thoracic vertebra using the simultaneous integrated boost technique at a dose of 25 Gy in 5 fractions and to the entire vertebral body in a dose of 35 Gy in 5 fractions (Figure 2). No toxicities were observed after treatment. The patient, who was 83 years old at the time this case was written, had a Karnofsky Performance Status score of 90% and complete urinary continence with only sporadic, weekly episodes of urinary urgency. The patient reported his sexual capacity to be satisfactory, having preserved the potentia coeundi capacity. No local recurrence was documented, resulting in a 100% local control rate. The patient has clinical follow-up and PSA control every 4 months.

## DISCUSSION

SBRT is an effective treatment for oligometastatic bone prostate cancer, resulting in persistent decreases in PSA level, and the possibility of treating new bone metastases with repeat SBRT has been described as an emerging treatment.^8-11^ The majority of studies show SBRT to be well tolerated among elderly patients with metastatic prostate cancer.^5-11^

This case involves the intriguing clinical scenario of a patient with bone oligometastatic prostate cancer who asked for personalization of therapy to avoid worsening his quality of life. When the patient presented in 2009, he refused standard-of-care ADT treatment because of the possible side effects, but few studies had been published about SBRT as an alternative to ADT. Nevertheless, the patient elected metastasis-directed SBRT at that time, and 6 additional courses of SBRT were administered during the subsequent 10 years for other bone oligometastases encountered during follow-ups.

After each course of SBRT, the patient's PSA level decreased. His prostate cancer was controlled at the most recent follow-up (last PSA value of 0.12 ng/mL), his Karnofsky Performance Status score was good, he had no treatment-associated toxicity, and he reported a satisfactory sex life.

The choice of metastasis-directed radiotherapy is now corroborated by several studies reporting that SBRT for oligometastatic bone prostate cancer is effective and well tolerated. Administering SBRT as early local therapy might delay the administration of ADT, which can cause a range of side effects (cardiovascular diseases, sexual dysfunction, osteoporosis with bone fractures, diabetes, and hot flashes) that can negatively affect quality of life.^7,12-14^

**Figure 3.**
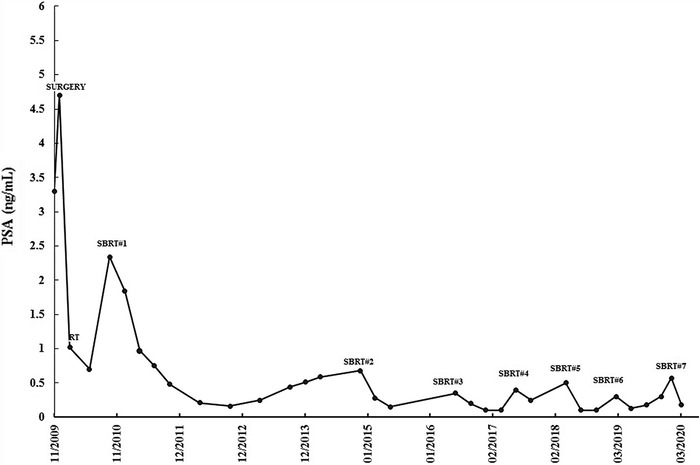
Prostate-specific antigen (PSA) levels at the time of the patient's surgery, prostate bed radiotherapy (RT), and 7 cycles of stereotactic body radiotherapy (SBRT).

The first-line imaging procedure for restaging prostate cancer recurrence is PET/CT with prostate cancer–specific PET radiotracers that can visualize tumors as small as 5 mm in diameter. According to the literature, C-11 choline and F-18 choline are the most-adopted radiotracers, although gallium-68 has been reported to be more sensitive at low PSA levels (<1 ng/mL).^15^ Our patient had repeat F-18 choline PET/CT imaging each time his PSA levels significantly increased, even if the PSA value did not reach 1 ng/mL (Figure 3). This approach was adopted initially because of patient anxiety but, over time, was continued because of the ability of PET/CT to diagnose new oligometastases at low PSA values. For our patient, the correspondence between PSA increase and PET/CT positivity was tight, so bone oligometastatic sites were treated with SBRT after each radiologic diagnosis.

Such close follow-up and the number of PET/CTs could be criticized because of the examination cost and possible patient-induced apprehension. However, we believe that the cost of ADT and the potential related toxicity would have resulted in a worse cost/efficacy balance in this case. For selected patients, repeated metastasis-directed SBRT is an effective alternative therapy that may maintain patient quality of life.

## CONCLUSION

Patients with bone oligometastatic prostate cancer can be treated with SBRT alone, and SBRT can be repeated when new oligometastases are encountered in other bone sites. For our patient, SBRT provided long-lasting palliation in the absence of relevant iatrogenic toxicity.

## Notes

*This article meets the Accreditation Council for Graduate Medical Education and the American Board of Medical Specialties Maintenance of Certification competencies for Patient Care and Medical Knowledge.*
